# Effect of conspecific neighbors on the foraging activity levels of the wintering Oriental Storks (*Ciconia boyciana*): Benefits of social information

**DOI:** 10.1002/ece3.6693

**Published:** 2020-08-30

**Authors:** Lei Cheng, Lizhi Zhou, Yiwei Bao, Nazia Mahtab

**Affiliations:** ^1^ School of Resources and Environmental Engineering Anhui University Hefei China; ^2^ Anhui Province Key Laboratory of Wetland Ecosystem Protection and Restoration (Anhui University) Hefei China; ^3^ Anhui Biodiversity Information Center Anhui University Hefei China

**Keywords:** conspecific behavior, foraging activity, foraging intensity, foraging neighbors, Oriental Stork, social information

## Abstract

Animals prefer to aggregate in patches with high abundance and availability of food resources. Group foragers typically receive information about food resources by monitoring external events and the behavior of neighbors. The Information Centre Hypothesis proposes that aggregations increase foraging activity levels as a result of social information provided by conspecifics. Increasing the foraging rate has as a result decreasing time devoted to anti‐predator vigilance and may intensify competition among group members. Studies have shown that foraging activities are influenced by factors other than flock size, such as the number and foraging intensity of neighbors. To test these hypotheses, we examined the effect of number and foraging intensity of neighbors on the foraging activity levels (foraging rate, foraging effort, and foraging success rate) of the wintering Oriental Storks (*Ciconia boyciana*). In this study, we collected focal sampling data on the foraging behavior of storks at Shengjin Lake during winter from 2017 to 2019, controlling the effects of other variables (group identity, wintering years, and wintering periods). We found that foraging activity levels were higher in the presence of foraging neighbors than in their absence. Moreover, individuals adjusted their foraging activity levels according to social information gathered from the behavior of neighboring conspecifics. Focal individuals’ foraging rate and foraging effort were positively correlated with the average foraging rate of neighbors. Their foraging success rate was not influenced by the average foraging rate and foraging success rate of neighbors; however, it was positively correlated with the average foraging effort of neighbors. In conclusion, foraging activity levels of individuals are primarily driven by the intensity of the foraging activity of neighbors. This result differs from the results of previous studies that suggested that flock size was the most important factor determining individual foraging activity levels.

## Graphical abstract

Our research focuses on the effect of conspecific behavior neighbors on the foraging activity levels of focal individual within a group. Previous studies have suggested that flock size is the most important aspect determining birds’ foraging activity levels. However, in this study, we show that the foraging behavior of individuals is in fact primarily driven by the foraging activity levels of neighboring individuals of the same species, and not only by flock size. Therefore, our work advances our knowledge about the foraging decision process of group animals.

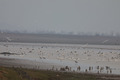

## 1 INTRODUCTION

Many animal species live in groups throughout their lives or during a certain annual lifecycle stage (Krause & Ruxton, 2002). Animal aggregation may provide information to conspecifics that enables these animals to make better decisions regarding foraging, habitat choice, and sharing of social information (Evans, Votier, & Dall, 2016; Gil, Hein, Spiege, Baskett, & Sih, 2018; Krause & Ruxton, 2002). Animals often use social information to obtain food resources (e.g., individuals or groups foraging in one patch alert others to take food), and to monitor and avoid predators (Dall, Giraldeau, Olsson, McNamara, & Stephens, 2005; Danchin, Giraldeau, Valone, & Wagner, 2004; Hein et al., 2015; Powell, 1974; Spiegel & Crofoot, 2016). Compared with independent solitary foraging, collaborative aggregation behavior may be a trade‐off response between food abundance and predation risk when animals foraging in a patch (Favreau et al., 2014; Møller & Laursen, 2019).

The Information Centre Hypothesis (Ward & Zahavi, 1973) states that aggregation increases foraging efficiency because of the social information provided by conspecifics. Information obtained in conspecific groups is typically about potential predators and food opportunities (Bekoff, 1996; Richner & Heeb, 1995). Initially, there were two possible explanations for foraging efficiency to be higher in aggregated animals: (a) with increasing flock size, individual animals can reduce the time devoted to anti‐predator detection and increase foraging rate (Lima, 1995; Lima & Dill, 1990; Lima, Zollner, & Bednekoff, 1999); (b) as flock size increases, competition between individuals searching for resources occurs more often, thus increasing their foraging rate (Beauchamp, 2005; Beauchamp & Livoreil, 1997). A significant number of ecologists have tested the accuracy of these two explanations in a large number of studies. Rieucau and Giraldeau (2009) tested whether increased foraging rate was the product of competition or anti‐predation when the flock size increased in Nutmeg Mannikins (*Lonchura punctulata*). The findings supported the idea that the increase in the foraging rate in groups is mainly due to the increased competition rather than the lower in predation risk. Therefore, increased competition due to aggregation could be responsible for the higher rate of foraging, as all individuals prefer to maximize their foraging effort (Favreau et al., 2014).

Communal habitats have the advantage of acting as information centres where individuals advertise and share information about the location of food sources (Ward & Zahavi, 1973). In these patches, the transfer of information is rapid and many individuals can be quickly attracted to form a successful forager flock (Barta & Giraldeau, 2001; Evans et al., 2016). However, when the flock size over a certain threshold, information transfer can be impeded by distance and position relative to other individuals (Fernández‐Juricic & Kacelnick, 2004; Beauchamp, 2015, 2017). In this situation, social foragers obtain information through their monitoring of external events and by monitoring their neighbors’ behaviors (Fernández‐Juricic & Kacelnik, 2004; Giraldeau & Caraco, 2000). Members of a group can benefit from the information on food location (local enhancement) and food quality (public information) being shared (Fernández‐Juricic, Erichsen, & Kacelnik, 2004), as well as from the presence of other individuals to determine when to enter and exit highly abundant but exposed foraging areas. Foraging decisions in groups are affected not only by the detection of food resources or potential predators by individuals but also by the behavior of conspecific neighbors (Beauchamp, 2003; Clark & Mangel, 1986; Galef & Giraldeau, 2001; Giraldeau & Beauchamp, 1999). As flock size increases, individuals can adjust their behavioral decisions based on social information acquisition, and this enables them to exploit the discoveries of other group members and minimize their costs (Dall et al., 2005; Fernández‐Juricic et al., 2004). Although previous studies have mostly focused on the effect of flock size on foraging activity levels (Collazo, Gilliam, & Miranda‐Castro, 2010; Gyimesi, Stillman, & Nolet, 2010; Maï, 2005), the behavior of conspecific neighbors is a source of information that may directly or indirectly affect the costs and benefits of social foraging (Beauchamp, 1998; Giraldeau & Caraco, 2000; Roberts, 1996).

The intensity of the behavior of conspecific neighbors may also affect foraging activity levels (Fernández‐Juricic & Kacelnick, 2004; McDougall & Ruckstuhl, 2018). In a sense, foragers use the behavior of group members to estimate food availability without needing to sample the whole patch (Clark & Mangel, 1986; Valone, 1989; Valone & Templeton, 2002). This monitoring reduces the costs of social foraging (Valone, 1989, 1993). Previous studies have shown that Starling (*Sturnus vulgaris*) and Great Tit (*Parus major*) can successfully use information obtained from neighbors and recognize when their foraging efforts are being successful (Krebs & Inman, 1992; Marchetti & Drent, 2000; Templeton, 1998). In addition to the individual actively receiving information from their neighbors, it turns out that behaviors are startlingly contagious between individuals in a group (Chartrand & Van Baaren, 2009; Chartrand & Bargh, 1999; Ginelli et al., 2015; McDougall & Ruckstuhl, 2018). Unlike imitation, contagious behaviors typically comprise instinctive behaviors that do not require learning (Zentall, 2003). Individual bird forages faster and more efficiently when accompanied by a group member than when alone (Hughes, 1971). It is showed that, when neighbors increased their foraging effort and success rate in conditions of high food availability, focal individuals also raised their foraging activities accordingly (Fernández‐Juricic & Kacelnick, 2004). Moreover, the detection of a neighbor that is successful at preying (e.g., with a high foraging rate) may promote an increase in an individual's foraging intensity (Smith, Benkman, & Coffey, 1999; Valone, 1993). As the number of neighbors and foraging rate increases, competition also increases. The food is depleted more rapidly in the short‐term (Maheswaran & Rahmani, 2001), and the chances of an individual obtaining food for themselves will decrease. Most studies have focused mainly on mammals, fishes, and forest birds (Fernández‐Juricic & Kacelnick, 2004; Fernández‐Juricic, Beauchamp, & Bastain, 2007; Gil et al., 2018; McDougall & Ruckstuhl, 2018), and there is little information about how foraging activity levels of waterbirds influence foraging decisions.

In the present study, we observed the foraging behaviors of the wintering Oriental Storks under different flock size conditions at Shengjin Lake (SJL), which is the important wintering site of Oriental Stork and lies on the south bank of the Yangtze River in Anhui Province, China (Cheng, Zhou, Wu, & Feng, 2020; Wang & Yang, 1995). This study aimed to understand how the number of neighbors foraging and the intensity of their foraging activity levels affected the foraging strategies of storks when neighbors were present. Based on the findings of previous studies, we tested three hypotheses: (a) foraging activity levels are higher in the presence of neighbors than in their absence; (b) the number of active neighbors foraging has a greater influence on individual foraging activity levels than the total number of neighbors; moreover, foraging rate, foraging effort, and foraging success rate increase with the number of neighbors foraging; (c) as neighbors show higher foraging intensity, the foraging rate and foraging effort of individuals increase simultaneously, whereas foraging success rate decreases with rapid depletion of food. We compared the significance of the effect of these variables and explored whether foraging activity levels of individuals are primarily driven by the foraging intensity of neighbors when neighbors are present.

## 2 MATERIALS AND METHODS

### 2.1 Study area and species

**Figure 1:**
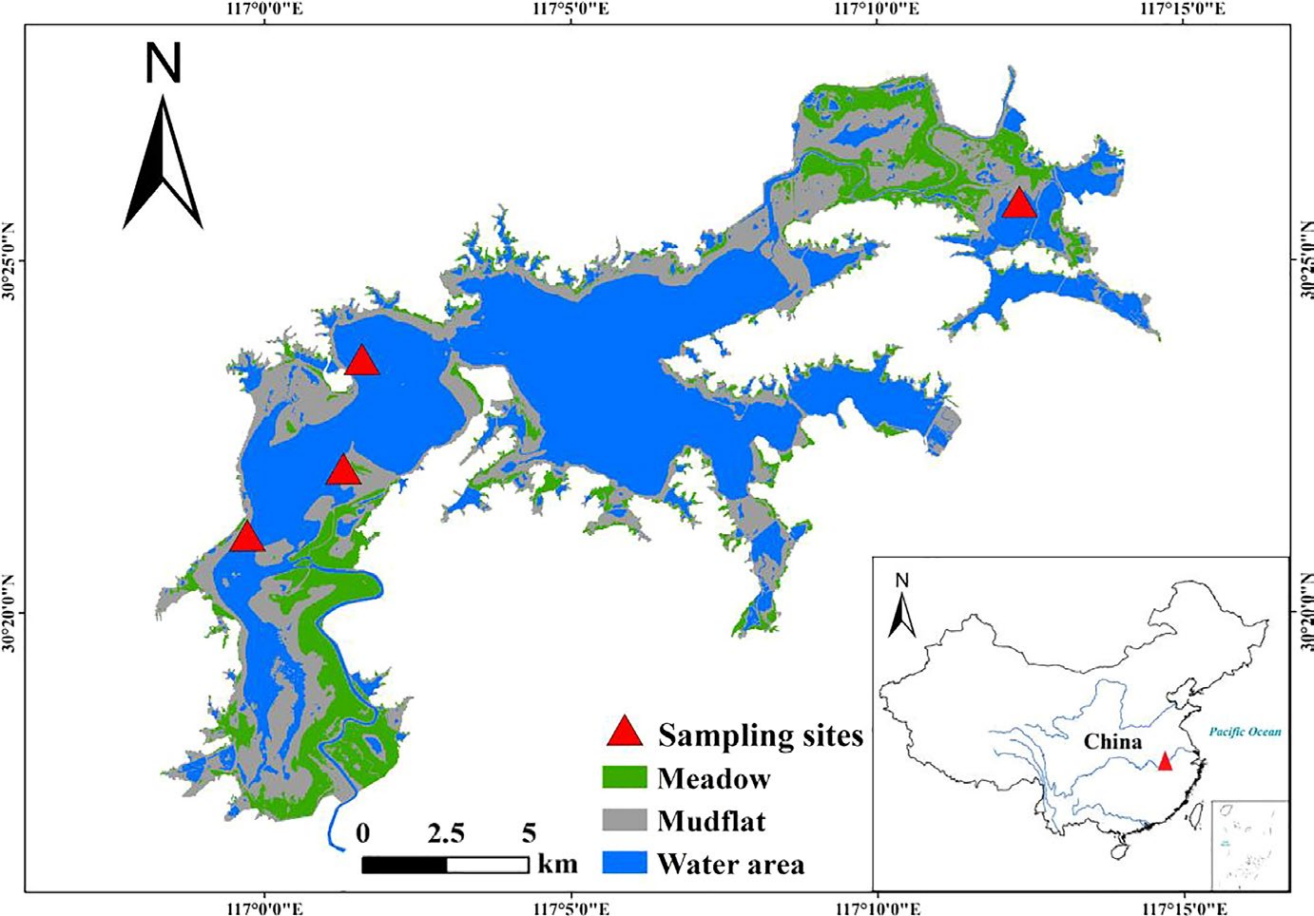
The major foraging sites of the wintering Oriental Storks at Shengjin Lake during the period from 2017 to 2019

Shengjin Lake (30°15′–30°30′ N, 116°55′–117°15′ E) is part of the lake–river complex of the Yangtze River floodplain and seasonally functions as the catchment area for the southern Anhui mountains (Figure 1). The total area of Shengjin Lake wetland is 16,800 ha, and the length of the lake shoreline is 165 km. It is an important stopover site and wintering ground for migrant waterbirds on the East Asian‐Australasian Flyway. This region has a subtropical humid monsoon climate, with average annual temperature and rainfall of 16.1°C and 1,600 mm, respectively. The Zhangxi and Tangtian rivers are the main tributaries of Shengjin Lake and run into the upper and central lakes, respectively. The foraging habitats of the wintering Oriental Storks are mainly in the upper and lower parts of Shengjin Lake, where most storks gather because of the abundant fishery resources. At Shengjin Lake, the main food resources for the Oriental Storks are fishes, such as *Cyprinus carpio*, *Carassius auratus*, *Pelteobagrus nitidus*, *Monopterus albus*, and other aquatic species (e.g., *Cipangopaludina sinensis*, *Cipangopaludina cathayensis*, and *Macrodrachium nipponensis*; Wang & Yang, 1995).

### 2.2 Data collection time and sites

We collected field data on the storks from 07:00 to 17:00 during two successive winters from November to March (2017–2018 winter and 2018–2019 winter). We observed the storks along the lake bank at four main points (Lianhe, Chi'an, Yang'etou, and Yanwo).

### 2.3 Measuring flock size and the number of neighbors

After locating a flock, the “look‐see” counting method was used to estimate flock size (Delany, 2005). Previous studies according to the individual's distance to the nearest neighbor as a standard measure to quantify the number of neighbors (Fernández‐Juricic & Kacelnik, 2004; Roberts, 1996). It is proposed that neighbors should be defined by a predetermined distance to the focal individual (Fernández‐Juricic et al., 2007). Within this distance, social information can be obtained or transmitted quickly by deliberate signals or inadvertent actions (Dall et al., 2005), thus allowing the animals to use the social information more effectively (Campobello & Sealy, 2011). In the present study, all individuals within a 10 m (approximately 10 body lengths) quadrat around the focal individual were considered to be neighbors. The value of 10 m was determined based on observations indicating that this was the typical distance between one focal individual and the most proximate neighbor (>90%, preliminary observation).

### 2.4 Measuring the water depth at foraging site

Water depth where individuals caught fish or foraged was roughly estimated using the bird's leg (tarsus + tibia) length (full leg approximately 70 cm) (Zbyryt, Sparks, & Tryjanowski, 2020). The water was considered to be shallow and deep when less than and more than 1/3 of the birds’ legs were underwater, respectively.

### 2.5 Behavioral observation

Behavioral observations were performed from a relatively hidden and reasonable distance place (usually behind bushes or slopes about 200 m away from the storks), measured with a laser rangefinder (Nikon 1200S with a range of 10–1,100 m). Before collecting behavioral data, we waited a few minutes for the group members to calm down and to ensure that the foraging activities of storks were proceeding without interferences. At each survey point, one foraging individual from the flock was randomly selected and observed. At the start of each sampling, we recorded the date, time, location, and water depth. A focal sampling technique comprising a 5 min duration to the observation of individual behavior events via a monocular telescope was used (SWAROVSKI 20–60 × 85, Absam, Austria) (Martin & Bateson, 1993), and behaviors were recorded on a mobile phone (storage capacity 256 G) connected to the monocular telescope through a converter (50–65 MM). The sampling was canceled if we lost sight of the focal individual. The observations concentrated on the foraging behavior of storks when they were searching, handling, and swallowing food. In this process, we recorded the following parameters: starting and ending time of each foraging bout, time spent foraging, time spent on locomotion, and the number of pecks. Flock size was determined at the beginning of the observations, and whenever the focal individual moved away from the group or flock size changed, the session was ended and a new observation bout was initiated. Later, during data processing, the videos were replayed and specific details of the foraging behavior were noted. We recorded a total of 123 video samples (100 group foraging video samples and 23 solitary foraging video samples). For group foraging, the mean value of flock size was 14.480 ± 0.948, the mean number of neighbors was 6.230 ± 0.350, and the mean number of neighbors foraging was 2.530 ± 0.134 (Table S1).

### 2.6 Statistical analysis

During the whole observation, if a stork caught a fish with its bill and then swallowed it, this meant they had foraging success. Through field observations and relevant literature, we used three variables to characterize foraging activity levels: foraging rate (the total number of pecks within a 1‐min period), foraging effort (the ratio of the total amount of time spent searching for and processing food and the activity time budget), and foraging success rate (the percentage of times that foraging was successful as a percentage of the total number of foraging behaviors; Amano, Ushiyama, Fujita, & Higuchi, 2006; Fan, Zhou, Cheng, Song, & Xu, 2020; Kuwae, Miyoshi, Sassa, & Watabe, 2010; Wan, Zhou, & Song, 2016). We defined the foraging activity intensity of neighbors as (a) the ratio of neighbors foraging, NFR = the number of neighbors foraging/the total number of neighbors × 100% and (b) foraging activity levels of neighbors.

We used PotPlayer (Version 1.7.21126, Kakao Corp.) to replay and analyze the videos on the computer with frame by frame viewing. The foraging activity levels data (foraging rate, foraging effort, and foraging success rate) of solitary foragers and group foragers had non‐normal distributions and were homoscedastic. Therefore, we used a nonparametric test (Kruskal–Wallis *H* test) to compare the foraging activity levels between solitary foraging (absent neighbor) and group foraging (present neighbor). Second, to analyze the effect of the number of neighbors and neighbors foraging on foraging activity levels of focal individuals, we chose the generalized linear model (GLM) analysis.

Finally, to understand the effect of the foraging intensity of neighbors on the foraging activity levels of the focal individual, we used a generalized linear mixed model (GLMM). Before the model analysis, we used the variance inflation factor (VIF) to assess whether the multicollinearity between explanatory variables and the low VIF values (<1.5) indicated that there was no significant difference. Then, we used a GLMM model with a binomial distribution (GLMMb) to test the effect of the foraging activity levels of neighbors on foraging effort and foraging success rate of focal individuals. The average foraging activity levels (average foraging rate, average foraging effort, and average foraging success rate) of neighbors were included in the model as fixed factors, and wintering years, wintering periods, and IDs as random factors. The foraging rate of the focal individual was tested using a GLMM model with a Poisson distribution (GLMMp). The average foraging activity levels (average foraging rate, average foraging effort, and average foraging success rate) of neighbors were included in the model as fixed factors and wintering years, wintering periods, and IDs as random factors.

All statistical analyses were performed in the R software 3.6.1 (R Core Team, 2019) and SPSS Statistics software 22.0 (IBM SPSS Statistics, 2013). A significance level of *p* = .05 was used for all statistical tests and results stated as Mean ± SE.

## 3 RESULTS

### 3.1 Effect of absence or presence of neighbors on individual foraging activity levels

**Figure 2:**
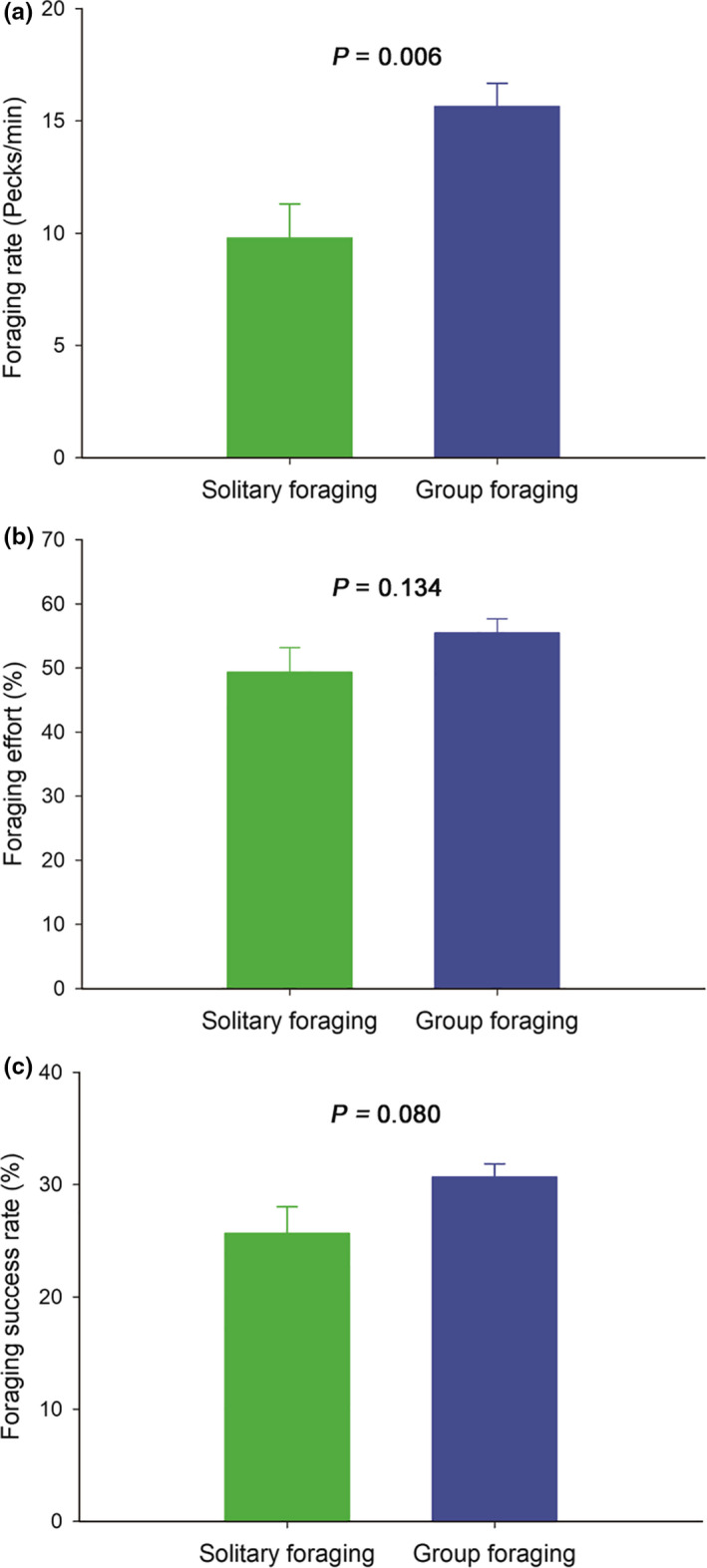
Comparison of foraging activity levels between solitary foraging (absent neighbor) and group foraging (present neighbor) of the wintering Oriental Storks. Error bars represent standard errors. (a) Foraging rate, (b) foraging effort, and (c) foraging success rate

Individuals with neighbors had higher foraging rates (15.687 ± 1.024 versus 9.797 ± 1.514, χ^2^ = 7.636, *p* = .006, Figure 2a), foraging efforts (55.472 ± 2.212 versus 49.352 ± 3.839, χ^2^ = 2.245, *p* = .134, Figure 2b), and foraging success rates (30.591 ± 1.200 versus 25.672 ± 2.340, χ^2^ = 3.068, *p* = .080, Figure 2c) compared with solitary foragers. This suggests a positive effect of neighbors on individual foraging activity levels.

### 3.2 Effect of the number of neighbors and neighbors foraging on foraging activity levels of focal individuals

**Figure 3:**
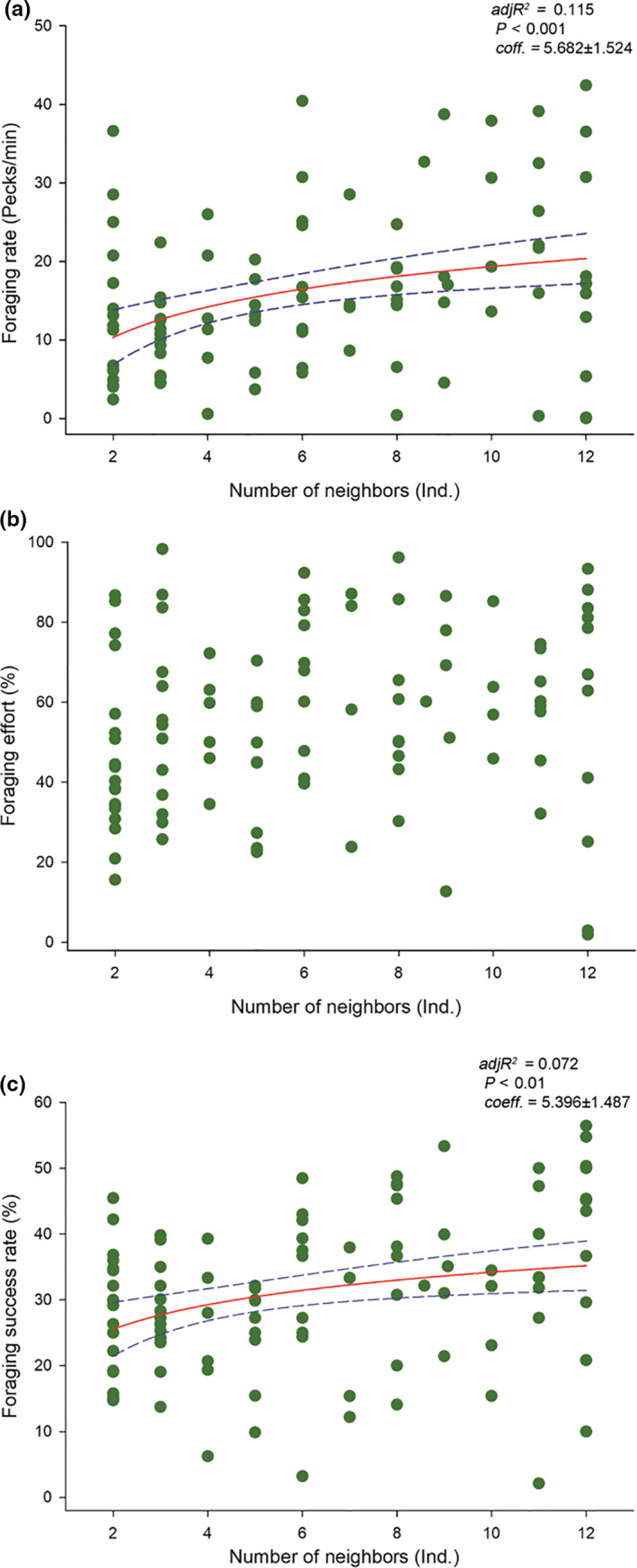
Effect of the number of neighbors on foraging activity levels of the wintering Oriental Storks. Each point represents a single sample, and the regression line ± the 95% confidence interval are shown as solid and dashed trend lines, respectively

**Figure 4:**
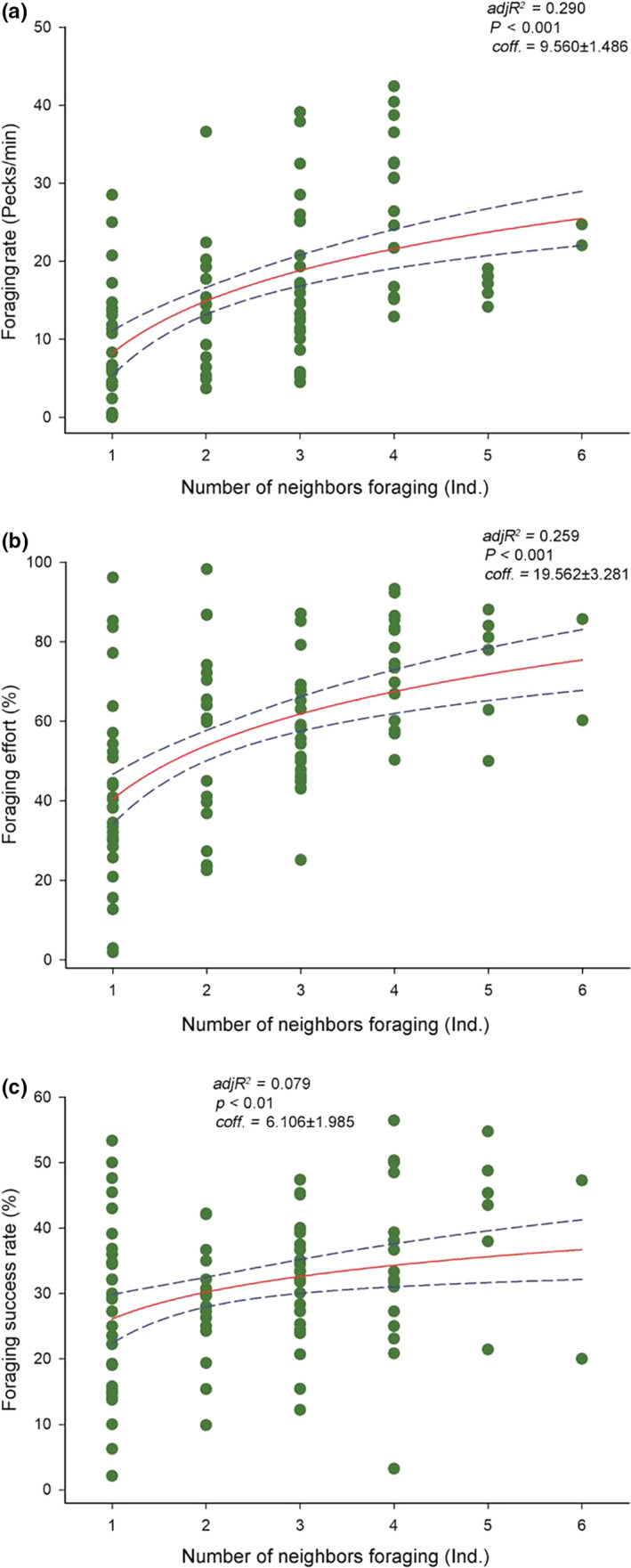
Effect of the number of neighbors foraging on foraging activity levels of the wintering Oriental Storks. Each point represents a single sample, and the regression line ± the 95% confidence interval are shown as solid and dashed trend lines, respectively

Foraging rate and foraging success rate of the focal individuals increased with the number of neighbors, whereas foraging effort was not correlated with the number of neighbors (Figure 3). However, foraging rate, foraging effort, and foraging success rate were correlated with the number of neighbors foraging (Figure 4). Furthermore, we found that individuals had a more significant and sensitive fit with the number of neighbors foraging than the number of neighbors (Figures 3 and 4).

### 3.3 Effects of foraging intensity of neighbors on the foraging activity levels of focal individuals

**Figure 5:**
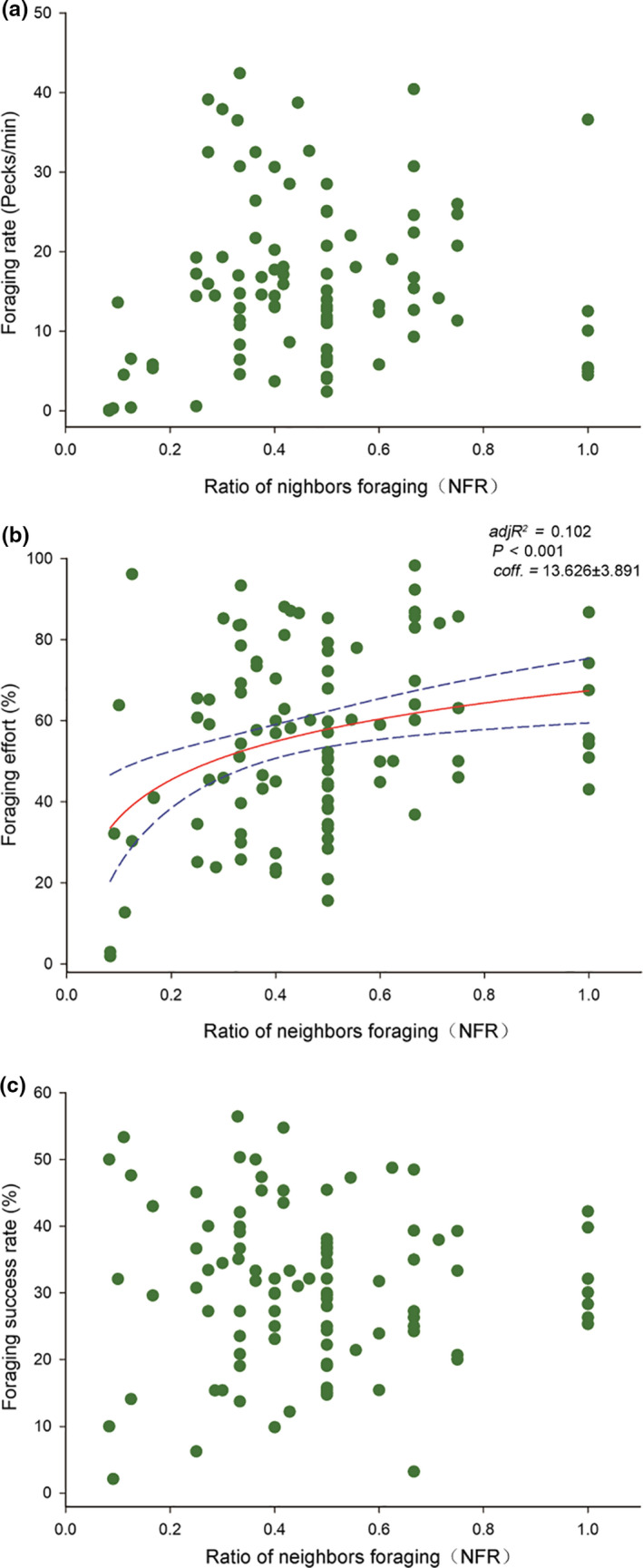
Effect of the ratio of neighbors foraging (NFR) on foraging activity levels of the wintering Oriental Storks. Each point represents a single sample, and the regression line ± the 95% confidence interval are shown as solid and dashed trend lines, respectively

The ratio of neighbors foraging (NFR) positively affected the foraging effort of focal individuals (*p* < .001, adjR^2^ = 0.102). However, foraging rate and foraging success rate were not correlated with the NFR (*p* = .083; *p* = .882) (Figure 5). Furthermore, we controlled for wintering years, wintering periods and flock IDs, the GLMM model showed that the average foraging rate of neighbors positively affected foraging rate and foraging effort of the focal individual (*p* = .041, coeff. ± SE = 0.009 ± 0.004; *p* < .001, coeff. ± SE = 0.063 ± 0.004) (Table 1), while the foraging success rate of focal individuals was significantly enhanced when the neighbors increased their average foraging effort (*p* < .001, coeff. ± SE = 2.187 ± 0.107) (Table 1).

**Table 1:** Results of the general linear mixed models (GLMM) testing whether foraging activity levels of the individual wintering Oriental Storks were related to neighbors of average foraging rate, foraging effort, and foraging success rate within a flock, controlled the effects of winter years, winter periods, and flock IDs

Foraging activity levels	Effects	Estimate value	SE	*Z*‐value	*p*‐value
Model 1: Foraging rate of focal individual	NF.Ave_FR	0.009	0.004	2.039	**.041**
	NF.Ave_FE	0.070	0.208	0.338	.736
	NF.Ave_FSR	−0.185	0.190	−0.969	.332
Model 2: Foraging effort of focal individual	NF.Ave_FR	0.063	0.004	17.845	**<.001**
	NF.Ave_FE	0.004	0.164	0.022	.983
	NF.Ave_FSR	0.298	0.146	2.039	**.040**
Model 3: Foraging success rate of focal individual	NF.Ave_FR	0.001	0.004	0.327	.744
	NF.Ave_FE	2.187	0.107	20.390	**<.001**
	NF.Ave_FSR	0.053	0.094	0.569	.570

Abbreviations: NF.Ave_FR, average foraging rate of neighbors; NF.Ave_FE, average foraging effort of neighbors; NF.Ave_FSR, average foraging success rate of neighbors.

## 4 DISCUSSION

Our results revealed that the foraging activity levels (foraging rate, foraging effort, and foraging success rate) of the wintering Oriental Storks were higher in the presence of neighbors than in their absence. Moreover, these levels were more influenced by the number of neighbors foraging than the number of neighbors, and the foraging activity levels increased considerably with the increased number of neighbors foraging. The first and second hypothesis, namely, (a) the foraging activity levels are higher in the presence of neighbors than in their absence, and (b) the number of active neighbors foraging has a greater influence on individual foraging activity levels than the total number of neighbors; moreover, foraging rate, foraging effort, and foraging success rate increase with the number of neighbors foraging, were confirmed. Finally, the foraging effort was significantly influenced by the ratio of neighbors foraging (NFR), but the foraging rate and foraging success rate were not. Furthermore, an increase in the average foraging rate of the neighbors promoted the foraging activity levels of focal individuals. Therefore, the third hypothesis (c) as the neighbors show higher foraging intensity, the foraging rate and foraging effort of individuals increase simultaneously, whereas the foraging success rate decreases with the rapid depletion of foods, was only partially supported.

### 4.1 Neighbors presence can provide benefits related to increased foraging activity levels

The presence of conspecifics influences an individual's behavior (Fernández‐Juricic & Kacelnick, 2004), and information transfer becomes less effective as group size increases (Ballerini et al., 2008; Beauchamp, 2015, 2017). Typically, individual foragers actively search for food, while neighbors follow successful foragers to feeding patches (Evans et al., 2016). In our study, the foraging activity levels of groups of storks were higher in the presence of neighbors than in their absence. Foraging groups locating food resources more easily than solitary foragers may be explained simply by the number of individuals that are available to search the habitat. Shared information on patch quality in a foraging group allows a rapid and accurate assessment of patch quality (Valone & Templeton, 2002). That is, the patch where animals gather tends to have a high abundance of food resources (Fretwell & Lucas, 1969). Furthermore, animals constantly enter and exit many patches before an aggregation is formed, so they spend time searching for highly productive foraging areas. As a result, an individual can gain energy by improving foraging activity levels after the aggregation is formed. Although group foragers never encounter patches exploited by others, they must, however, pay the cost of having to search for new patches more often than solitary foragers (Krause & Ruxton, 2002). Group foraging remains the most efficient strategy to exploit resources as it allows groups to avoid areas partly or entirely explored earlier by other individuals, thereby increasing the foraging success rate (Miller, 1922).

### 4.2 The number of neighbors foraging significantly affects individual foraging activity levels than the total number of neighbors

The importance of the number of neighbors in the foraging behavior of an individual bird has been previously reported (Smith, 2002; Templeton, 1998). However, subsequent studies have pointed out that it is more effective for foragers to learn from the behavior of conspecifics when estimating food availability (Valone & Templeton, 2002; Fernández‐Juricic & Kacelnick, 2004). We found that the influence of the number of neighbors foraging on the foraging activity levels of the storks had a greater effect than the total number of neighbors. Meanwhile, the foraging activity levels had a more significant and sensitive fit with the number of neighbors foraging. Storks may spend less time estimating food abundance and availability when there are more foraging neighbors because the patches are selected by more individuals. As the number of neighbors foraging increases, individual storks may increase their foraging rate and foraging effort to maximize energy gain and reduce the loss of foraging opportunities (Maheswaran & Rahmani, 2001; Zhao, Lyu, Sun, & Zhou, 2019). Our results indicate that the number of neighbors foraging positively affected focal individuals, which can be explained by the transfer of behavior, that is, when foraging with neighbors, individuals will actively increase their foraging activity levels to reduce the risk of energy shortfall (Smith et al., 1999). In a manner, greater flock size and foraging neighbors mean more individuals competing for food. This could be another reason that storks increase foraging activity levels to search for the next food item (Fortin, Boyce, Merrill, & Fryxell, 2004).

Besides, foraging rate also varies with individual factors, such as sex, age, personality, and physiological conditions (Domènech & Senar, 1999; Ruckstuhl, Festa‐Bianchet, & Jorgenson, 2003; Bergvall, Schäpers, Kjellander, & Weiss, 2011; Favreau et al., 2014). The researchers considered that between individual variation exists in the trade‐off between foraging rate and vigilance (Carter, MacDonald, Thomson, & Goldizen, 2009; Dannock, Blomberg, & Goldizen, 2013; Favreau et al., 2014; Nussey, Wilson, & Brommer, 2007). The relationship between foraging and personality has been tested in some animals, and studies have shown that bold individuals forage for longer and exhibit higher foraging rates than shy ones (Bergvall et al., 2011; Carter, Goldizen, & Tromp, 2010; Kurvers et al., 2010). Besides, Waite (1987) found differences in vigilance and foraging rate between the sexes in birds. Although individual factors affected the trade‐off between foraging rate and vigilance, it was difficult to collect data from our research subjects in the field. Banding storks to identify them in future studies would help solve this question.

### 4.3 The foraging activity levels of individuals are primarily driven by the foraging intensity of neighbors when neighbors are present

When the foraging intensity of neighbors increased, the focal individual also enhanced its foraging activity accordingly. That is, these storks are influenced by the foraging behavior of their neighbors. In the present study, foraging effort improved as the ratio of neighbors foraging (NFR) increased, but this did not affect the foraging success rate. The likely explanation is the changes in food abundance and availability after water depth has decreased, that is, the source, distribution, location, and size of food resources may importantly affect the variation in foraging success rate (Evans, Inta, Lai, & Lenz, 2007; Gill, 2007; Wilson, 1978). Consequently, variation in food abundance and availability should be considered in future studies to satisfactorily clarify the relationship between foraging activity levels and the number of neighbors foraging. The foraging rate was also not influenced by the ratio of neighbors foraging (NFR) as well. The possible reason is that this ratio transfers information about the abundance and availability of food resources, rather than on the foraging speed of other neighbors.

To better understand the influence of the foraging intensity of neighbors on the foraging activity levels, we used a GLMM for further analysis. The results of this analysis showed that the increase in the average foraging rate of neighbors promoted the foraging activity levels of the focal individual and that the foraging success rate of focal individuals was significantly affected by the average foraging effort of the neighbors. On the one hand, storks may adopt a conservative foraging strategy by lowering the foraging rate and foraging effort when foraging alone. Lowering the foraging rate can reduce the energy cost by reducing frequent head down movements to search for food in the water. Additionally, the lower foraging rate allows a longer time gap for searching for food by visual techniques. While foraging in groups, the storks adopt a relatively radical foraging strategy by increasing the foraging rate and foraging effort. This may be explained by the fact that the aggregation of animals may be related to the existence of a larger quantity of food resources, and storks improve their foraging activity levels to increase chances of obtaining food using tactile techniques and rapid searches. An alternative interpretation, which also considers the use of social information, is that the response of a focal individual may be caused by the competition between foraging neighbors (Beauchamp, 2003; Clark & Mangel, 1986). As the foraging intensity of neighbors increases, the competition also increases, and food is depleted more rapidly in the short‐term (Maheswaran & Rahmani, 2001). Therefore, individuals might increase their foraging activity levels to prevent neighbors from monopolizing food resources or displacing them from food patches.

## 5 CONCLUSION

Taken together, our results indicate that the foraging activity of storks was higher in the presence of neighbors than in their absence. The wintering storks adjusted their foraging activity levels according to social information gathered from conspecific individual neighbors. Foraging rate and foraging effort were positively correlated with the average foraging rate of neighbors. Foraging success rate did not change with the average foraging rate and foraging success rate of neighbors, but it was positively correlated with the average foraging effort of neighbors. Therefore, this study revealed that individual foraging activity levels are primarily driven by the foraging intensity of neighbors. Previous studies have considered flock size to be one of the most important factors in determining individual foraging activity levels. However, our results showed that the foraging activity levels of the wintering Oriental Storks in flocks were significantly affected by the foraging activity of neighbors. Social transmission of information about food resources or danger is transmitted not only within species but also between nearby individuals of different or distantly related species. Therefore, in future studies, we will focus on questions such as how neighbors foraging of different species can influence the foraging activity of individuals in mixed‐species groups; in addition, we will determine how individuals adjust their foraging behavior based on visual information obtained from the body posture of neighbors.

## CONFLICT OF INTEREST

None declared.

## AUTHOR CONTRIBUTION

**Lei Cheng:** Data curation (lead); Formal analysis (equal); Investigation (lead); Methodology (equal); Visualization (equal); Writing‐original draft (lead); Writing‐review & editing (lead). **Lizhi Zhou:** Conceptualization (lead); Resources (lead); Supervision (equal); Visualization (equal); Writing‐review & editing (equal). **Yiwei Bao:** Investigation (supporting); Writing‐review & editing (supporting). **Nazia Mahtab:** Methodology (supporting); Writing‐original draft (supporting).

### OPEN RESEARCH BADGES

This article has earned an Open Data Badge for making publicly available the digitally‐shareable data necessary to reproduce the reported results. The data is available at Supporting Information Table S1.

## Supporting information

Table S1

## Data Availability

Data are provided as Table S1.
